# Simultaneous MRI and laser Doppler Flowmetry: Assessing cerebral Macro- and microcirculation in neurointensive care

**DOI:** 10.1016/j.nicl.2025.103821

**Published:** 2025-06-16

**Authors:** Sofie Tapper, Stina Mauritzon, Marcelo P. Martins, Fredrik Ginstman, Anders Tisell, Peter Zsigmond, Karin Wårdell

**Affiliations:** a Department of Biomedical Engineering, Linköping University, Linköping, Sweden; b Department of Radiology in Linköping and Department of Health, Medicine and Caring Sciences, Linköping University, Linköping, Sweden; c Department of Neurosurgery in Linköping and Department of Biomedical and Clinical Sciences, Linköping University, Linköping, Sweden; d Department of Medical Radiation Physics and Department of Health, Medicine, and Caring Sciences, Linköping University, Linköping, Sweden; e Center for Medical Image Science and Visualization (CMIV), Linköping University, Linköping, Sweden

**Keywords:** 2D flow MRI, Arterial Spin Labeling (ASL), Cerebral Blood Flow (CBF), Subarachnoid hemorrhage (SAH), Vasospasm, Neurocritical care, Optical probe

## Abstract

**Objective:**

Subarachnoid hemorrhage (SAH) patients are monitored in the neurointensive care unit (NICU) to avoid additional brain injuries, yet methods for monitoring cerebral blood flow (CBF) are limited. The aim was to investigate the feasibility of simultaneous MRI, using arterial spin labeling (ASL) and 2D-flow MRI, and probe-based laser Doppler flowmetry (LDF) in NICU patients.

**Method:**

Four SAH patients, three of whom received optical probes during routine surgery, were included. Compatibility of simultaneous LDF and MRI was evaluated before performing MRI 3–4 times over several days per patient. The methods were compared using mean CBF in grey matter obtained from the absolute ASL CBF-maps, the total inflow calculated from 2D-flow MRI, and local average LDF perfusion. Additionally, regional mean CBF from ASL and each arterial flowrate was compared for each hemisphere.

**Result:**

Twelve MRI measurements were successfully performed, eight of which included simultaneous LDF. With careful routines and the MR scanner uniquely located in the NICU, neither patient safety nor data quality was compromised, demonstrating the feasibility of concurrent measurements. All methods showed longitudinal dynamic changes, following the same increasing or decreasing trends. In three patients, ASL and 2D-flow data were closely related, with dynamic changes within 10 %.

**Conclusion:**

This novel approach offers longitudinal, simultaneous estimates of macro- and microcirculatory components locally, regionally, and globally in the human brain. This concept has potential to provide insights into the interplay of different aspects of CBF in NICU patients and thereby aid in prevention of secondary brain injuries.

## Highlights

• The first study of simultaneous MRI and LDF monitoring of cerebral circulation in humans.
• Longitudinal measurements were achieved in four critically brain-injured patients.
• Total inflow, global and regional CBF, and local perfusion were quantified.
• Patient safety and data quality were maintained during simultaneous measurements.
• This approach highlights both macro- and microcirculation in neurocritical care.

## 1 Introduction

Subarachnoid hemorrhage (SAH) is a type of cerebral bleeding defined as the extravasation of blood into the subarachnoid space, often resulting from a traumatic brain injury or ruptured aneurysm (Abraham and Chang, 2016). Poor-grade SAH, which is associated with high morbidity and mortality, presents significant challenges in patient management and outcomes (Claassen and Park, 2022). One hypothesis is that these poor outcomes are related to the initial hemorrhage, triggering pathophysiological events that, within 3–14 days, may progress into vasospasm, delayed cerebral ischemia (DCI), or brain infarction (Geraghty and Testai, 2017, OSGOOD, 2021, Neifert et al., 2021). Therefore, SAH patients are often routinely and carefully monitored in the Neurointensive Care Unit (NICU) for up to 14 days, necessitating multifaceted care strategies to prevent secondary brain injury (Claassen and Park, 2022).

In the NICU, the monitoring approach is usually multimodal, including intracranial pressure (ICP), brain tissue oxygenation, and occasionally brain metabolism using microdialysis (MD) (Vitt et al., 2023). Monitoring cerebral blood flow (CBF) is crucial to detecting hypoperfusion and administering preventative treatment in time to avoid ischemia (Rasulo et al., 2018). Current best practice includes using daily transcranial Doppler (TCD) ultrasonography to measure the CBF velocity in the middle cerebral artery (MCA) (White and Venkatesh, 2006, KOFKE, 2023). Further examinations may include computed tomography (CT) perfusion and angiography. However, monitoring CBF is not straightforward, and a clinically available method for both continuous and spatially resolved CBF monitoring does not exist. Also, since macro- and microcirculation generally behave differently and may uncouple also in the brain in times of severe illness (Khanna and Karamchandani, 2021), simultaneous investigations of these components are highly valuable.

Optical methods (Durduran, 2014, Rejmstad et al., 2018) have been suggested for continuous, real-time, microvascular CBF monitoring. Laser Doppler flowmetry (LDF) (Nilsson et al., 1980) has been used in several NICU studies (Meyerson et al., 1991, Bolognese et al., 1993, Kirkpatrick et al., 1994, Mauritzon et al., 2022) with different optical probe designs. Our novel two-channel LDF probe can be implanted bilaterally alongside other standard monitoring equipment during routine surgery, thus avoiding extra intervention (Mauritzon et al., 2021). The main advantage of LDF adapted for cerebral tissue is the ability to provide bedside continuous estimates of the microvascular CBF with a high temporal resolution (Wårdell et al., 2024). However, the measurement is invasive and localized, and the two-channel probe can sample from a maximum of two locations.

Many imaging techniques for CBF measurements have evolved over the years (Hillman et al., 2005, KOFKE, 2023). Magnetic resonance imaging (MRI) is a non-ionizing radiation technique offering large spatial coverage at a high resolution. Using MR-based techniques, blood flow velocities can be assessed in larger vessels using 2D phase-contrast flow measurements (Wymer et al., 2020) and whole-brain perfusion estimates can be acquired using arterial spin labeling (ASL) (Detre et al., 1992, Iutaka et al., 2023, Williams et al., 1992). However, MRI provides only snapshot measures in time, limiting the practicality in the NICU, where a continuous need for CBF feedback is desirable.

A multimodal approach is required to capture variations over time of the various aspects of macro- and microcirculatory CBF components locally, regionally, and globally. One strategy is to utilize the continuous mode and high temporal resolution of microcirculation monitoring with LDF, and the high-resolution and extensive spatial coverage of CBF components provided using MRI techniques such as ASL and 2D-flow MRI. Workflows for repeated ASL (Tapper et al., 2024) and continuous LDF monitoring in the NICU (Mauritzon et al., 2022) have previously been implemented separately. The aim of this study was to implement and evaluate the feasibility of simultaneous measurements with ASL and 2D-flow MRI protocols and probe-based LDF in patients monitored in the NICU following an SAH. This work also included comparative analyses of the methods using longitudinal measurements to explore how different components of CBF measurements can complement each other in real clinical scenarios. Following MRI safety mitigation, the feasibility was evaluated in four patients with SAH undergoing neurocritical care.

## 2 Materials and methods

**Fig. 1:**
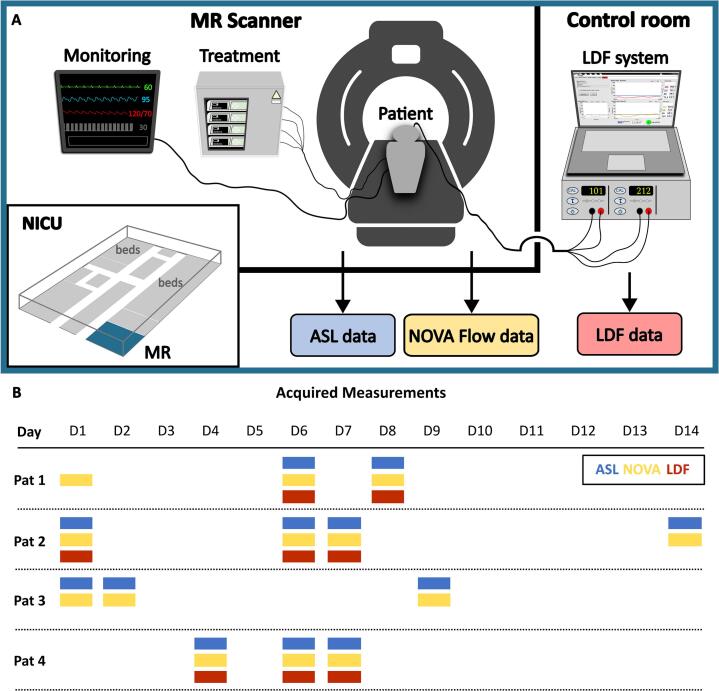
Overview of the measurement setup and data acquisitions. **A.** Measurement setup with the LDF system placed in the MRI control room during data acquisition. The MR scanner is located in the NICU, and monitoring and treatment needed throughout the MR measurements were performed with MR-compatible equipment. **B.** Patients (Pat) were scanned several times during the NICU stay, with D1 indicating day 1 of NICU arrival. For each patient, MRI measurements (ASL and 2D flow using NOVA) were performed with simultaneous LDF monitoring, when possible, as indicated and color-coded in the figure.

At the University Hospital in Linköping, a unique setup is available with an MRI scanner located in the NICU ward. This enables repeated MRI measurements of CBF during patient monitoring, significantly reducing the risks associated with patient transportation (Martins et al., 2025). In this paper, we expand this setup with fiber-optical microcirculation measurements using LDF. Fig. 1 presents an overview of the measurement situation. Details on the LDF system, MRI acquisition, patient measurements, and subsequent data analysis are provided below.

### 2.1 Laser Doppler flowmetry

Microcirculatory data were obtained using an LDF system (PF5000, Perimed AB, Sweden) previously adapted and evaluated for neurointensive care monitoring (Rejmstad et al., 2018, Mauritzon et al., 2022) (Fig. 1A). A two-channel flexible fiber-optical probe (Mauritzon, 2021) was designed for bilateral neurointensive care monitoring and inserted through burr holes during routine surgery. The surgeon aimed at positioning the probe in the frontal areas about one centimeter into the brain parenchyma. After insertion, the probe was secured with sutured wings to minimize motion artifacts. In addition to microvascular perfusion (*Perf*), the LDF system provides the total light intensity (*TLI*), reflecting the grey-whiteness of the tissue. Both measures are relative and given in arbitrary units (a.u.), ranging from 0 to 1000 a.u. and 0–10 a.u., respectively. The *TLI* was used during probe insertion to distinguish between the darker cortex and the brighter areas indicating subcortical matter, and further on during monitoring to assess possible changes in the probe location. Software for data acquisition was written in LabView (National Instruments, USA). The LDF time constant was set to 0.03 s. Patients were monitored for 4–6 h per day, supervised by a project member logging timestamps of specific events.

### 2.2 MRI acquisitions

MRI data were collected using a clinical 3 T MAGNETOM Skyra MR system (Siemens Healthineers, Erlangen, Germany) equipped with a 20-channel head coil (Fig. 1A).

#### 2.2.1 MRI safety

To ensure MRI safety and compatibility, the fiber-optical LDF probe was evaluated by an experienced MR physicist (AT). The probe was scanned in a water phantom, and image quality was evaluated on both a dynamic EPI and a T1w MPRAGE volume.

A risk analysis was done for the simultaneous use of optical probes, LDF, and MRI. Risks were evaluated from a usage and logistics perspective when adding the LDF system to the MR facilities, including risks for the patient as well as the healthcare staff.

During the MRI, the LDF system was placed in the control room as pictured in Fig. 1A. A 5 m optical cable connected the LDF system and the patient through a wave trap. All other equipment needed during MRI data collection was used according to the manufacturer’s instructions.

#### 2.2.2 Arterial spin labeling

A workflow for the acquisition, visualization, and quantification of repeated ASL MRI data has been previously published, including data from Patient 1 (Tapper et al., 2024). This workflow produces CBF maps in absolute units (ml/100 g/min), between measurement day. In addition to pulsed ASL images (scan time = 4 min 59 s., perfusion mode = FAIR QII, TE/TI/TI1/TR = 15.56/1990/700/4600 ms, FOV = 205 x 205 mm^2^, acquired resolution = 3.25 x 3.20 x 3.00 mm^3^, reconstructed resolution = 1.6 x 1.6 x 3.0 mm^3^, base resolution = 64, bandwidth = 2694 Hz/px, 3D GRASE readout, EPI factor = 21, turbo factor = 20, number of slices = 40), high-resolution 3D MPRAGE T1-weighted images (T1w, scan time = 5 min 21 s, TE/TI/TR = 2.29/900/2300 ms, flip angle = 8 deg., FOV = 240 x 240 mm^2^, slice thickness = 1 mm, voxel size = 0.9 x 0.9 x 1.0 mm^3^). Proton density-weighted (PDw, scan time = 2 min 22 s, TE/TR = 8.6/10000 ms, flip angle = 160 deg, FOV = 205 x 205 mm^2^, slice thickness = 3 mm) turbo spin echo images were acquired during each MRI session with a resolution agreeing with the ASL images. The T1w images were used for image registration and tissue characterization, and the PDw images for scaling to absolute units. Further details of the ASL MRI acquisition are found in (Tapper et al., 2024).

#### 2.2.3 2D flow MRI

The volume flow rate in the larger arteries was acquired using the Non-invasive Optimal Vessel Analysis (NOVA) software (VasSol, Chicago, USA), a commercially available software tool that uses a 3D model approach for 2D MR flow measurements (Zhao et al., 2000). Following a vessel scout over the head, a 3D MR angiography time-of-flight data was acquired (TE/TR = 3.42/21 ms, flip angle = 18°, slice thickness = 0.5 mm, matrix size = 580). These images were used to generate a 3D surface rendering of the brain’s vasculature. This 3D rendering could be freely rotated, allowing the operator to optimally select a measurement point along a straight vessel segment where laminar flow was expected. Using NOVA’s line-fitting algorithm (Zhao et al., 2000), the operator’s click was converted into coordinates corresponding to a plane perpendicular to the flow in the targeted vessels. These coordinates were subsequently transmitted to the MR scanner, where cardiac-gated 2D phase-contrast MR angiography (TE/TR = 6/110 ms, flip angle = 25°, slice thickness = 4 mm, matrix size = 256) was used to perform blood volume flow measurements for each selected vessel. The NOVA software automatically verified the velocity encoding to prevent aliasing of high velocities. If aliasing occurred, the scan was repeated for the specific vessel using the new proposed velocity range. Volumetric flow rates (ml/min) were acquired from the following arteries: bilaterally from the left and right anterior cerebral artery (ACA), MCA, posterior cerebral artery (PCA), internal carotid artery (ICA), vertebral artery (VA), and from the basilar artery (BA). In case of blood vessel anomalies or surrounding blood, measurements of specific vessels were excluded.

### 2.3 Patient measurements

Four patients were included in this study. Oral and written informed consent was obtained from their next-of-kin, as the patients were unconscious. The study was approved by the Ethics Review Board (EPN 2021–03527). Inclusion criteria were invasive monitoring, such as external ventricular drainage (EVD), ICP monitoring, and MD catheters, required from the start or expected within the first days. Patients must also meet general MR safety requirements. Table 1 summarizes the patient characteristics, and Fig. 1B summarizes the days of acquired data (ASL, NOVA, and LDF) per patient. A summary of each patient’s history is found below.

**Table 1:** Summary of the characteristics of the included patients. The main diagnosis was subarachnoid hemorrhage (SAH), in one case followed by an intracerebral hemorrhage (ICH). In addition to standard ICU monitoring, all patients were monitored using external ventricular drain (EVD) and microdialysis (MD), and three patients received laser Doppler flowmetry (LDF).

**Patient**	**Age**	**Sex**	**Diagnosis**	**Monitoring Devices**
1	47	F	SAH, ICH	EVD, MD, LDF
2	53	F	SAH	EVD, MD, LDF
3	57	M	SAH	EVD, MD
4	58	F	SAH	EVD, MD, LDF

#### 2.3.1 Patient 1

Patient 1 suffered an SAH following a right MCA aneurysm rupture, followed by a right hemispheric intracerebral hemorrhage. After hematoma evacuation, the patient was in the NICU for 11 days using standard monitoring. On day 4, the two-channel LDF probe was implanted bilaterally. On days 6 and 7, worse clinical status was observed, and border zone ischemia was suspected in the areas around the evacuated hematoma. Although no other obvious signs of DCI were present, induced hypertension was started with norepinephrine, and clinical status improved onward. MRI was done on three occasions: on day 1 with only NOVA flow measurements and on days 6 and 8 with simultaneous MRI (ASL and NOVA) and LDF (Fig. 1B).

#### 2.3.2 Patient 2

Following an SAH from a basilar tip aneurysm, Patient 2 was monitored in the NICU for 14 days. The two-channel LDF probe was implanted bilaterally on day 1. However, the channel on the right side failed to produce any signal. By day 6, although no clinical signs of DCI or vasospasm initially, spontaneous hypertension and increased TCD values prompted a CT perfusion later during the day. The CT perfusion showed signs of right-sided watershed hypoperfusion areas confirmed by conventional catheter angiography, which were not reversible with intra-arterial nimodipine. Induced hypertension was started with norepinephrine on the evening of day 6, which continued until day 10. On day 11, new signs of vasospasm developed, and induced hypertension was restarted until day 15, combined with endovascular treatment with nimodipine. Ischemic injuries were seen on morphological MRI on day 14. This patient was scanned four times with simultaneous MRI and LDF, except on day 14 when the LDF probe had been removed (Fig. 1B).

#### 2.3.3 Patient 3

Patient 3 suffered an SAH from an anterior communicating artery aneurysm and was monitored in the NICU for 14 days after endovascular coiling. No LDF probe was implanted due to accessibility reasons. Discrete signs of DCI encouraged induced hypertension from day 5 to day 10. Morphologic MRI showed subacute ischemic injury that might have appeared at the primary aneurysm rupture. MRI scanning was done on days 1, 2, and 9.

#### 2.3.4 Patient 4

Patient 4 suffered an SAH from an anterior communicating artery aneurysm, which was rebleeding at referral to the NICU. After aneurysm coiling, the LDF probe was inserted together with standard monitoring. On day 4, vasopressor doses were kept high to keep blood pressure at an adequate level, partly due to propofol sedation. On day 5, high TCD values in the left hemisphere prompted CT perfusion, which did not raise any concerns about vasospasm. On days 6 and 7, TCD values were normal, and the patient was hemodynamically stable onward.

### 2.4 Data processing and analysis

**Fig. 2:**
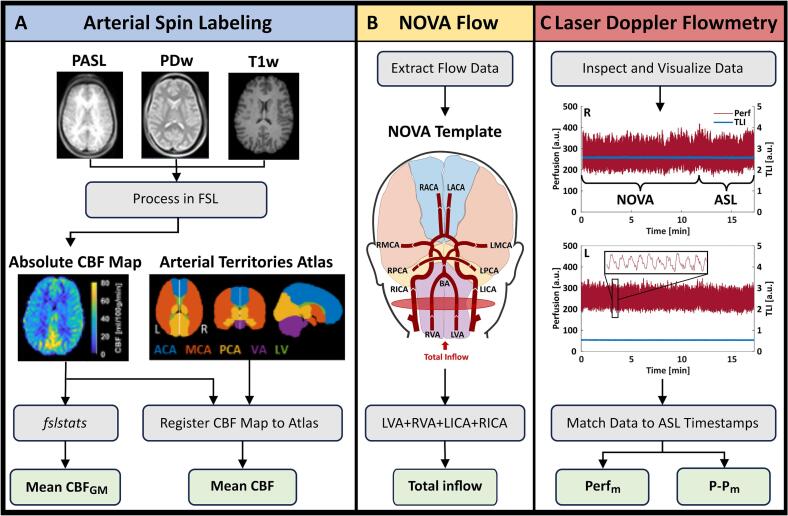
Overview of the acquired data, processing steps, and the calculated parameters for each technique. **A.** Pulsed ASL (PASL), proton density-weighted (PDw) and T1w images are needed to compute the CBF map in absolute units. This map was quantified using fslstats, generating an estimate of the mean CBF in grey matter (CBF_GM_), and registered to the arterial territories atlas, generating estimates of the mean CBF in each arterial territory. **B.** Each arterial flow rate (ml/min) was visualized using the NOVA template. The total inflow to the brain was calculated from the left and right vertebral (LVA, RVA) and internal carotid arteries (LICA, RICA). The ASL atlas is shown in the NOVA template to highlight the connection between each supplying artery and its corresponding territory. **C.** LDF data recorded during the ASL measurement was used to compute the mean perfusion (Perf_m_) and peak-to-peak (P-P_m_) in the right (R) and left (L) hemispheres, respectively.

Fig. 2 summarizes the processing and analysis steps performed on the ASL, NOVA, and LDF data. In addition, each probe location was identified on T2w images by tracking the path from the burr hole to the probe tip. Postoperative CT images, where MD catheters are visible, were used to distinguish those from the LDF probe. A physician (FG) determined the arterial territory for each probe location, which was marked on the T1w images overlaid with the atlas.

#### 2.4.1 ASL data

Processing of ASL data was done using functions from the FMRIB Software Library (FSL, v6) (Chappell et al., 2011, Jenkinson et al., 2012), adhering to the guidelines outlined in (Alsop et al., 2015). After processing the repeated MRI data, the resulting absolute CBF maps were registered to a patient-specific reference space based on the T1w images acquired during each patient’s first MR session. From each absolute CBF map, an estimate of the mean CBF in grey matter (GM, *CBF*_GM_), was obtained using the FSL function *fslstats*, with a voxel considered as pure GM if the partial volume estimate was ≥ 80 % GM (Asllani et al., 2008), (Chapell et al., 2023). Additionally, the quantification of regional *CBF* was performed using a digital 3D brain MRI arterial territories atlas (Fig. 2A) (Liu et al., 2023). The atlas was registered to the patient-specific reference space before calculating the regional mean *CBF*_._

#### 2.4.2 NOVA data

The NOVA flow data was extracted and visualized as shown in Fig. 2B. The total inflow to the brain was calculated by adding the flow rates in the left and right ICA and VA. Direct measurements of blood flow in the left and right ACA, MCA, and PCA were compared to the corresponding mean *CBF* in each arterial territory obtained from the ASL data. The atlas is overlaid in the NOVA template presented in Fig. 2B to highlight the connection between the different arteries and the corresponding arterial territories.

#### 2.4.3 LDF data

For the analysis of LDF data, only data recorded during the 5-minute ASL MRI were considered in this paper. These sections of data were extracted using the timestamps and protocol notations. From each section, the mean perfusion *(Perf*_m_) and the mean peak-to-peak (*P-P*_m_) were calculated for each channel (Fig. 2C).

#### 2.4.4 Comparison between methods

Changes in CBF were compared to evaluate the consistency between the three methods. This comparison was performed separately for each patient. Since these techniques measure different aspects of CBF and are expressed in different units, each series of measurement data was normalized to the results obtained from the first day of simultaneous measurements. This normalization allowed for the calculation of percentage changes, which were compared across the techniques. Normalized changes were calculated for the whole brain (*CBF*_GM_), the total inflow to the brain, and the LDF data, and for each arterial territory and the supplying arterial flow in the left and right hemispheres, respectively.

## 3 Results

**Fig. 3:**
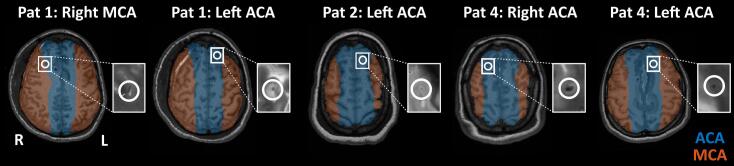
LDF probe locations, determined by a physician (FG) and visualized on the T1w images overlaid with the arterial territory atlas. For Patient 1, the LDF probe was placed in the watershed area between the MCA and ACA, leaning more toward MCA, in the right hemisphere and the ACA territory in the left hemisphere. Patient 2 only had one LDF channel, which was located in the left ACA territory. Patient 4 had the LDF probe in the ACAs in both the right and left hemispheres. The zoom-ins next to each T1w image highlight the visible marks from the probes.

Four SAH patients were scanned repeatedly over several days. Three patients (1, 2, and 4) received the optical LDF probe, and probe positions were confirmed on T2w images (Fig. 3). The feasibility of simultaneous measurements, measurement results, and comparison between the techniques is presented below.

### 3.1 Feasibility of simultaneous MRI and LDF

The LDF probe showed no image artifacts in the phantom measurements. Based on the risk assessment, a few key points were added to the MRI routine for this study: 1) one person is responsible for the LDF system, placing it at a designated spot in the MR control room, and not interfering with the clinical measurement routine, 2) the LDF probe cable should be placed leaving a clear path for healthcare staff to access the patient whenever needed, and 3) in case of emergency, a member of the MR staff should cut the LDF probe cable by the wave trap with an MR-safe scissors, and the LDF system should directly be removed from the area. Before patient measurements were conducted, evacuation training with the updated routine was performed for engineers, radiographers, and NICU staff.

Following these routines, simultaneous MRI and LDF recordings were highly feasible from both patient safety and data acquisition perspectives. No disturbances were observed in either the MRI data or the LDF data recorded during the MRI measurements, indicating that both techniques can be reliably used together without compromising data quality.

### 3.2 Patient measurements

**Fig. 4:**
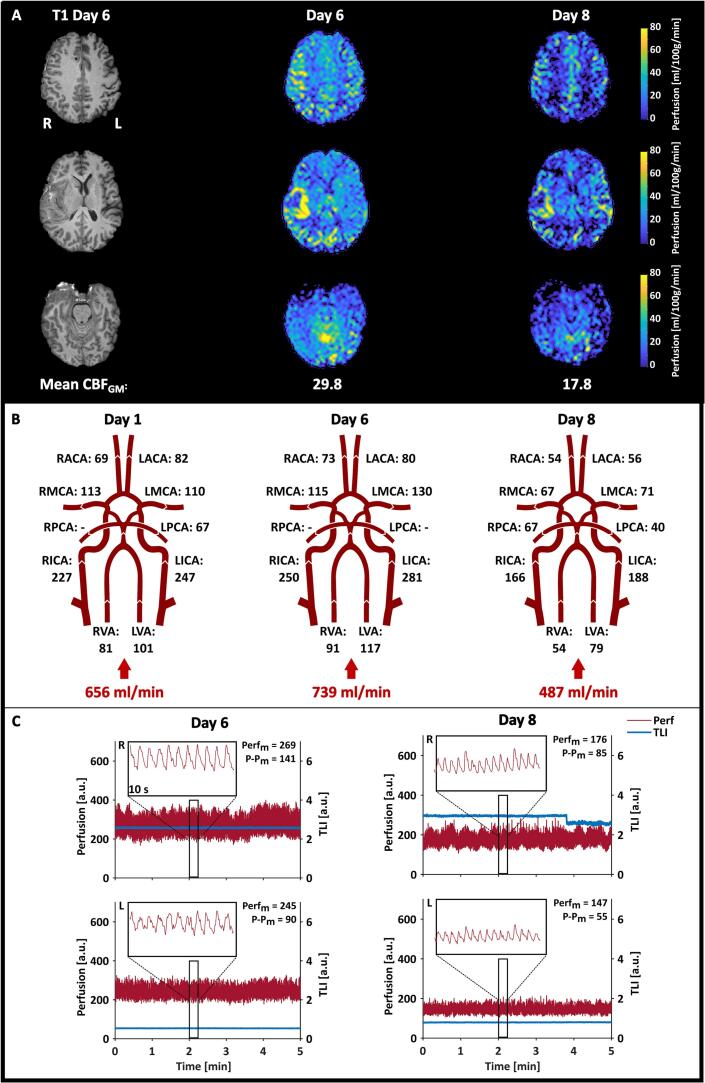
Summary of the results from the measurements performed on Patient 1. **A.** Absolute CBF maps calculated from the ASL data are shown in three sections. The resulting mean CBF_GM_ is depicted below each day. **B.** The resulting volumetric flow rates (ml/min) obtained from the NOVA flow data, with the calculated total inflow to the brain. **C.** The LDF data recorded during the 5-minute ASL MRI measurement in the left (L) and right (R) hemispheres. 10-second zoomed-in sections are also depicted to show the characteristics of the perfusion signal. The resulting Perf_m_ and P-P_m_ are given in each graph.

The measurement results for Patient 1 are summarized in Fig. 4. The absolute CBF maps revealed hyperperfused areas in the perihemorragic zone around the evacuated intracerebral hematoma on the right side (Fig. 4A). The initial NOVA flow measurement performed on day 1 indicated that the CBF was relatively stable between days 1 and 6 (Fig. 4B). In the LDF data, the *P-P_m_* values of the perfusion followed the same decreasing pattern as the rest of the data. Finally, the *TLI* remained relatively stable, except for a quick drop observed just before minute 4 on day 8 on the right side. Similar changes in the *TLI* have been observed over short periods previously during bedside monitoring and were not considered connected to the MRI examination.

**Fig. 5:**
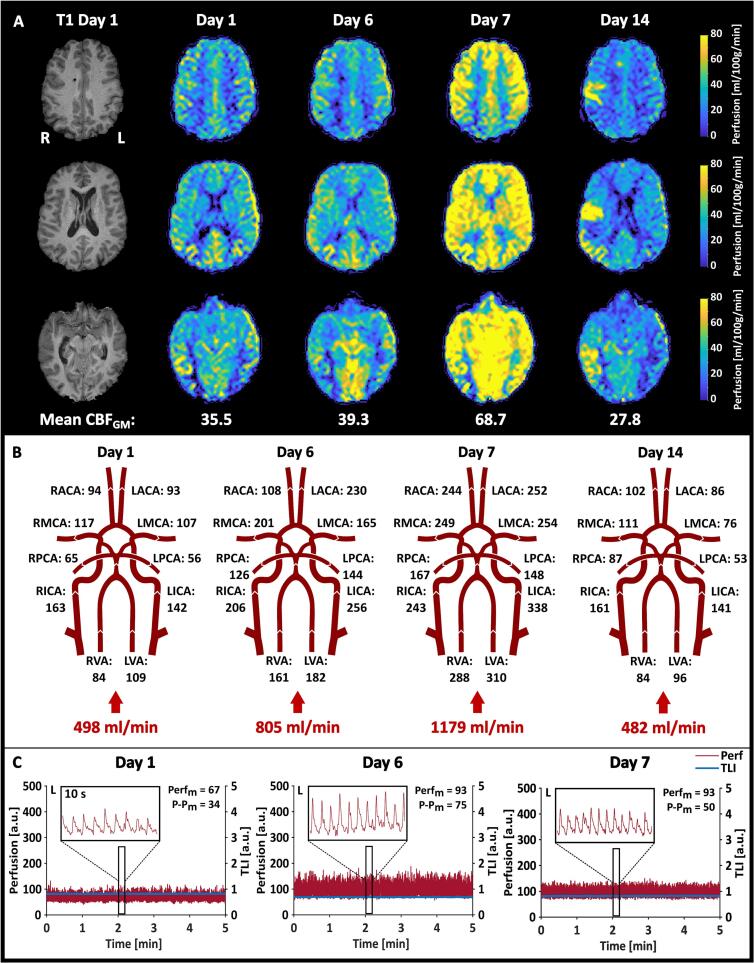
Summary of the results from the measurements performed on Patient 2. **A.** Absolute CBF maps calculated from the ASL data are shown in three sections. The resulting mean CBF_GM_ is depicted below each day. **B.** The resulting volumetric flow rates obtained from the NOVA flow data, with the calculated total inflow to the brain. **C.** The LDF data recorded during the 5-minute ASL MRI measurement in the left (L) hemisphere. 10-second zoomed-in sections are also depicted to show the characteristics of the perfusion signal. The resulting Perf_m_ and P-P_m_ are given in each graph.

Fig. 5 shows the results from the measurements performed on Patient 2. The absolute CBF maps revealed a completely hyperperfused map on day 7 and hyperperfused areas in the right hemisphere on day 14 around ischemic injuries seen on morphological MRI. The increase observed on day 6 stood out in the NOVA flow data (Fig. 5B). The LDF probe placed in the left hemisphere increased from day 1 to day 6, but instead of further increasing on day 7, like the MRI data, it leveled out (Fig. 5C). The pulsativity (*P-P*_m_), however, decreased from day 6 to 7. The mean *TLI* remained stable across all three measurement days, indicating a stable probe position.

**Fig. 6:**
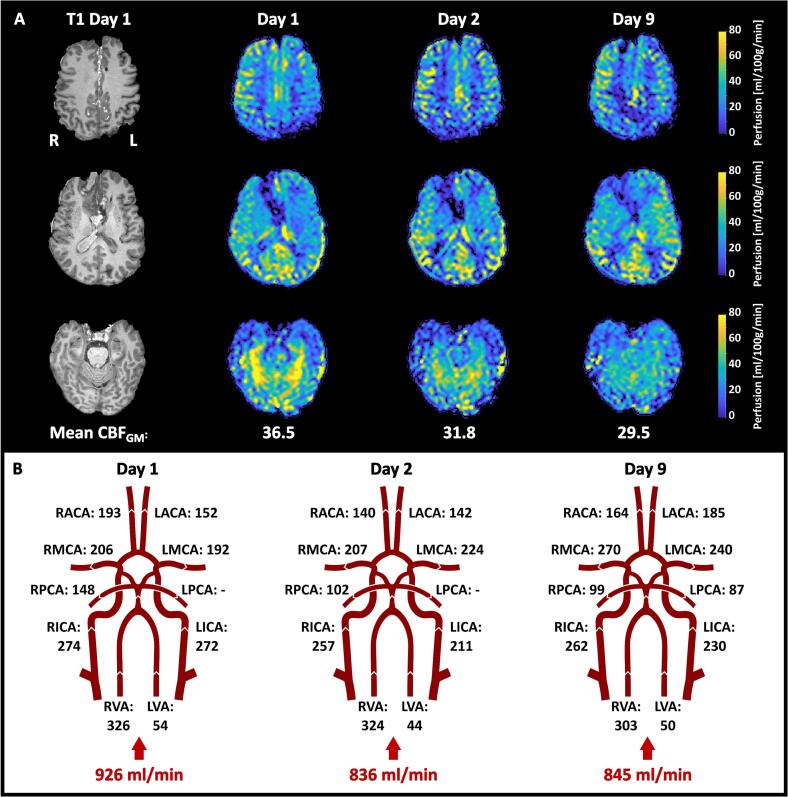
Summary of the results from the measurements performed on Patient 3. **A.** Absolute CBF maps calculated from the ASL data are shown in three sections. The resulting mean CBF_GM_ is depicted below each day. **B.** The resulting volumetric flow rates obtained from the NOVA flow data, with the calculated total inflow to the brain.

**Fig. 7:**
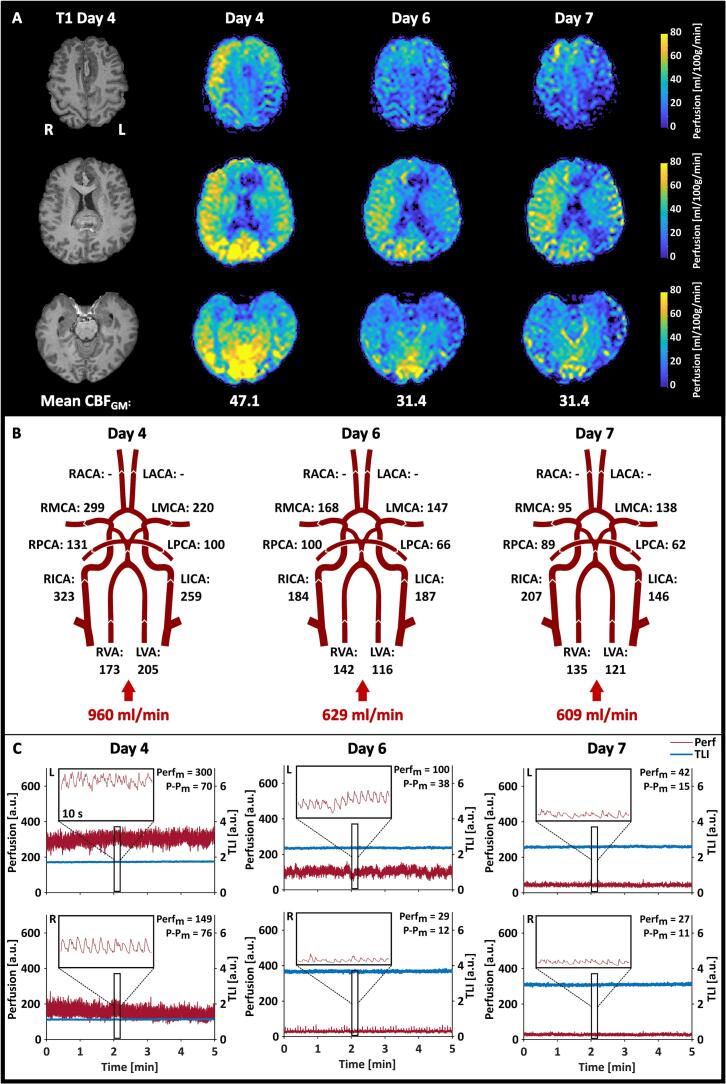
Summary of the results from the measurements performed on Patient 4. **A.** Absolute CBF maps calculated from the ASL data are shown in three sections. The resulting mean CBF_GM_ is depicted below each day. **B.** The resulting volumetric flow rates obtained from the NOVA flow data, with the calculated total inflow to the brain. **C.** The LDF data recorded during the 5-minute ASL MRI measurement in the left (L) and right (R) hemispheres. 10-second zoomed-in sections are also depicted to show the characteristics of the perfusion signal. The resulting Perf_m_ and P-P_m_ are given in each graph.

Fig. 6 shows the results from the repeated MR measurements performed on Patient 3, and Fig. 7 summarizes the results from Patient 4. The ASL maps for Patient 4 showed hyperperfused areas in the right hemisphere on day 4, which reduced over the days. The *TLI* remained stable in the left hemisphere but changed extensively in the right hemisphere between days 4 and 6, suggesting a change in probe position.

### 3.3 Comparison of measurement techniques

**Fig. 8:**
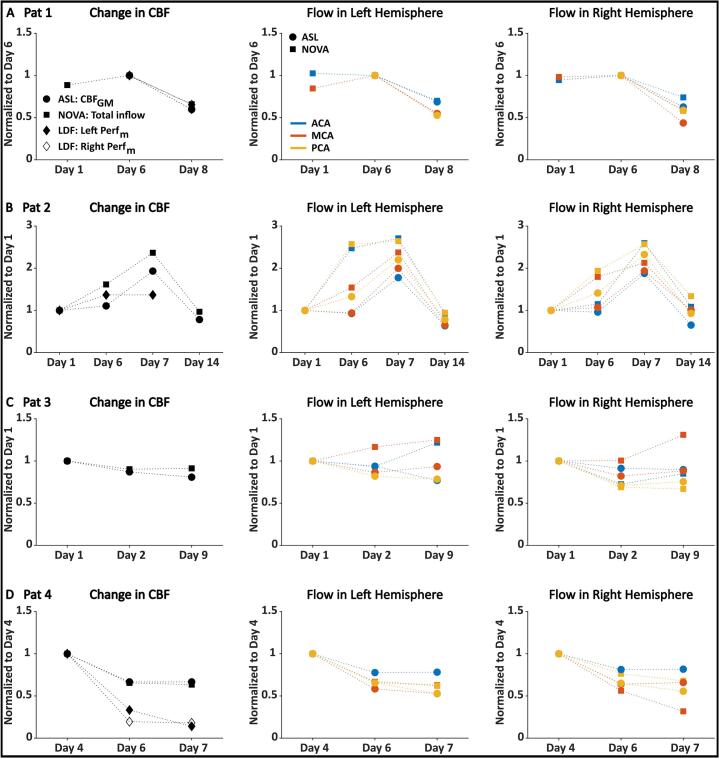
Comparison between the CBF measurement techniques. The first column shows normalized CBF changes in mean CBF_GM_, total inflow to the brain, and local mean perfusion, Perf_m_, for the right and left hemispheres, if applicable. The second and third columns show regional mean CBF quantified using the arterial territories atlas, compared to the blood flow in the corresponding supplying artery, for the left and right hemispheres, respectively. All data were normalized to the first day of simultaneous measurements, which differed for each patient and is stated on their respective Y axes. **A.** Data from Patient 1 (Pat 1), who had two LDF channels. The data was normalized to day 6 since only NOVA flow data was obtained on day 1. **B.** Data from Patient 2 (Pat 2), who only had one LDF channel in the left hemisphere. Note the higher range on the Y axis. **C.** Data from Patient 3 (Pat 3), who never received an optical probe. **D.** Data from Patient 4 (Pat 4), who had two LDF channels.

The normalized changes in CBF were compared between the methods and are summarized in Fig. 8. The data were compared for the whole brain (first column) and regionally in the left and right hemispheres (second and third columns).

In Patient 1, there was a clear decrease in CBF (Fig. 8A) between days 6 and 8 for all three measured parameters (ASL: −40 %, NOVA: −34 %, LDF: −35 % and −40 % for the right and left channels, respectively). Considering regional changes in CBF using the arterial territory atlas for the ASL data and the volumetric flow rates in the corresponding supplying artery for the NOVA data, the results showed that the CBF decreased in all territories in both hemispheres.

For Patient 2, all three methods showed an increase in CBF between days 1 and 6 (ASL: +11 %, NOVA: +62 %, LDF: +39 %). Between days 6 and 7, the MRI data continued to increase (ASL: +94 %, NOVA: +137 %, from day 1), while no percentage change was observed in the LDF data. However, the CBF increased more with NOVA flow than ASL, with the highest spread observed on day 6 in both the left and right hemispheres. Both the mean *CBF*_GM_ and total inflow to the brain decreased between days 7 and 14 to levels below day 1 (ASL: –22 %, NOVA: −3%).

Patient 3 showed a reduction from day 1 to 2 (ASL: −13 %, NOVA: −10 %), and slight changes in separate directions to day 9 (ASL: −19 %, NOVA: −9%) (Fig. 8C). However, the MCAs and left ACA stand out and appear to be the major recipients of the slightly increased total inflow observed between days 2 and 9. This increase was reflected regionally in MCA territories, but not in the left ACA territory.

For Patient 4 (Fig. 8D), all three methods decreased considerably from day 4 to day 6 (ASL: –33 %, NOVA: −35 %, LDF: −67 % and −81 % for the left and right sides, respectively), and remained at similar levels on day 7, except for the left LDF channel that continued decreasing (−86 %). The regional changes in the left and right hemispheres followed the global trend of remaining stable between days 6 and 7, except for the flow in the right MCA, which decreased further. This was, however, not reflected in the right MCA territory, which remained at the same level.

## 4 Discussion

A novel concept of repeated and concurrent MRI and LDF measurements of CBF in patients undergoing neurocritical care was introduced and demonstrated in four patients diagnosed with SAH. Comparison between CBF measured with ASL and 2D-flow from 12 MR sessions showed percentage changes in similar ranges, while greater variation was observed in the microcirculatory LDF data. All methods, however, revealed distinct variations over the days, showing how dynamic blood flow variations are in these patients. This concept enables studies of simultaneous cerebral macro- and microcirculation trends in severely ill patients.

### 4.1 Simultaneous MRI and laser Doppler flowmetry

Each technique has strengths and limitations, and effective monitoring should capture pathophysiological events before they cause irreversible brain damage, specifically before CBF drops below 10–12 ml/100 g/min (von Kummer and Dzialowski, 2017). In this study, concurrent MRI and LDF were suggested, as this combination can measure CBF globally using 2D flow and the NOVA software for rendering the vessel tree, globally and regionally using ASL, and locally and continuously using LDF, without apparent interaction between the methods. To our knowledge, this is the first time the feasibility of simultaneous MRI and LDF has been demonstrated in humans. Experimental studies, however, exist. The prediction of ischemic outcome in rats was investigated using LDF monitoring intra-procedurally during MCA occlusion, followed by perfusion-weighted MRI (Cuccione et al., 2017). Although concluding a good agreement between LDF monitoring and MRI data, simultaneous MRI and LDF were not performed. Another group (He et al., 2007) found strong correlations between percentage responses in ASL data and superficial cortical LDF measurements when studying functional changes in CBF in anesthetized rats. In line with our results, this study highlights the feasibility of combined measurements without compromising the data quality of either technique.

Patient safety is another important aspect to consider in terms of feasibility. Patient safety during the MRI process, including the transportation method, has already been evaluated in another study (Martins et al., 2025) with promising results. By addressing the risks associated with adding the LDF system to the procedure and establishing careful routines and training for staff involved in simultaneous measurements, no complications were introduced. However, the patient’s general medical condition and individual risk factors must be evaluated before every MR scan, since they may vary during the NICU course.

### 4.2 Patient measurements

Thirteen MRI sessions were successfully performed in four patients, of which twelve included both ASL and NOVA, and eight of those included simultaneous fiber-optical LDF measurements in one or two spatial locations. The resulting measurement trends followed each other, but the data showed variability between the patients and were therefore considered individually.

The two MRI methods were more closely related to each other than to the LDF measurements. This was expected since they assess CBF from different perspectives and at different spatial resolutions. Percentage changes within 10 % were found for the ASL and NOVA measurements for Patients 1, 3, and 4 (Patient 1: −34 to −40 %, Patient 3: −9 to −19 %, Patient 4: –33 to −37 %) over all days. The ASL and NOVA data for Patient 2 showed the same increasing trend, but increased more with NOVA initially, which was not reflected in the ASL maps on day 6. A higher total inflow measured by NOVA theoretically results in a higher overall CBF measured by ASL. An altered flow, distribution, or dispersion of blood within the brain could imply changes in the patient’s condition. For instance, a large increase in CBF or uncoupling between total inflow and distribution within the brain might indicate an upcoming event, such as the one observed in Patient 2 on days 6 and 7. The increased total inflow and the hyperperfused maps seen on day 7 are likely a reflection of the induced hypertension treatment started in the evening on day 6. Still, the increased total inflow on day 6 had no obvious clinical explanation.

Microcirculation was monitored with LDF over many days in three patients, adding another perspective on the CBF and its variations over time. In our calculations of the *Perf*_m_ and *P-P*_m_, only the 5-minute window during the ASL sequence was considered. The LDF results followed the increasing or decreasing trends observed in the MRI data, but varied in intensity. In Patient 1, the LDF was in line with the MRI measurements (−35 to −40 %), while in Patient 2, the LDF increase (+39 %) was in between the ASL (+10 %) and NOVA (+62 %) and remained at this level when the ASL and NOVA measurements increased further (to + 94 % and + 137 %, respectively) until the next day. We have already discussed the discrepancy observed between the ASL and NOVA data on day 6, but the lack of response in the LDF data on day 7 may be another interesting marker. While succeeding in increasing the total inflow with induced hypertension, an increase in the microvascular blood flow still failed. This is one very important aspect, since if lacking a microvascular effect, the macrovascular changes are without purpose. In complex cases, having additional information from LDF may be beneficial. Yet, with only one example, no conclusions can be drawn from this data. In Patient 4, the LDF measurements showed a larger decrease of around −67% to -86 % compared to the –33 % to-37 % decrease observed in the MRI data. However, in one of the LDF channels, the *TLI* changed remarkably between the first and the following days, suggesting that the probe position had changed, thus not monitoring the same subset of blood vessels. The increase in *TLI* indicates brighter tissue, which in the brain means approaching white matter with a generally lower blood flow than in grey matter. This may explain the decrease observed in the LDF channel placed in the right hemisphere and should be considered in the interpretation.

Another aspect that could explain the discrepancies between ASL and LDF data is that the mean *CBF*_GM_ was calculated for the whole brain and the different arterial territories, thus containing global and regional CBF information, whereas the LDF data is local. While the macro- and microcirculatory components belong to the same circulatory system, they do not necessarily follow the same patterns because of their distinct roles and regulatory mechanisms (Moore et al., 2015) and deviations in critical illness (Khanna and Karamchandani, 2021). In microcirculation, local metabolic demand causes spatial and temporal variations, which are not necessarily reflected in the mean calculations of ASL over larger areas. Consequently, a strong correlation between the two measures cannot be assumed in all cases. In contrast, the ASL and LDF correlation study by He et al. (He et al., 2007) showed a strong correlation between ASL and cortical LDF data. In their study, the ASL results were calculated based on the volume around the LDF probe, and in that case, a stronger correlation can be expected.

Further measurements are needed to explore the relationships between these methods and by doing so, learn more about the macro- and microcirculatory interplay in brain injury patients. With more data, discrepancies between the methods and their relevance for neurointensive care can be evaluated. A second track worth investigating is whether these methods could complement each other in neurointensive care. Ideally, the continuous mode of LDF could give earlier warnings of deterioration in the microcirculation and thus indicate when another MRI examination is necessary to assess the patient’s CBF status extensively over the whole brain. This may be feasible with bilateral LDF monitoring achieved over longer periods, providing both local and, to some extent, global information. Additionally, this setup would allow for validation of the LDF data by comparing it to the ASL data in the same volume, and comparative studies could increase the knowledge about patterns to look out for in the LDF data. Given the distinct variations observed over time in this data, these methods have the potential to track dynamic changes in CBF longitudinally.

### 4.3 Limitations and Future work

The analysis of MR and LDF measurement data presents some challenges. Firstly, these three measurement techniques assess various aspects of CBF at different spatial and temporal resolutions. For example, there is a large variation in perfusion depending on whether the measurement is performed in grey or white matter. Recent studies using LDF have shown that the perfusion can change from one millimeter to another (Wårdell et al., 2024). The regional perfusion values were calculated using the arterial territories atlas, which was not further modified to solely incorporate contributions from different tissue types, to minimize sources of errors. Secondly, each technique produces results in different measurement units. This diversity complicates the interpretation of the results, especially when contradictions occur. While using percentage changes can address the issue of differing measurement units, this approach introduces difficulties when baseline measurement data is unavailable. Other methodological concerns include ensuring stable NOVA flow measurement points throughout the longitudinal MR measurements, as well as maintaining consistent LDF probe locations between and during the MRI. In our LDF measurements, a stable *TLI* indicates that the probe is well-fixated and not moving. It is well known that the perfusion signal is sensitive to external movements. Due to the good fixation of the probes during implantation, we received an artifact-free perfusion signal even during MR scanning. However, given the *TLI* change in one channel in Patient 4, the probe had likely moved slightly, and thus, we could not guarantee that the same position was being monitored over the days. By using a cranial skull bolt, the fixation of the probes may improve. Another limiting factor is the small number of patients included. To validate our results, a larger patient group is needed. As a proof-of-concept study, the comparison between the methods was limited to mean calculations of the resulting data. With more data available, data variability should be considered for each method. As a next step, we plan to include more SAH patients and add patients with traumatic brain injuries. In another ongoing study, the LDF data collected at the bedside are investigated and compared to clinical data such as mean arterial pressure and ICP.

Additionally, clinical progression can vary widely between patients, influenced by the severity of the hemorrhage and individual recovery processes. Not all patients require invasive monitoring devices implanted during routine surgery, and therefore, an LDF probe cannot be implanted in these cases. Furthermore, the presence of MR-unsafe devices and clinical status may exclude individuals from MR examinations. Thus, it is not always possible to combine these methods.

As a final point, performing MR measurements on patients in a clinical environment presents challenges and opportunities compared to measurements on healthy controls. This is particularly true in the context of neurointensive care, where continuous monitoring and treatment must be maintained throughout data acquisition.

## 5 Conclusion

This proof-of-concept study demonstrates the feasibility of longitudinal and simultaneous MRI and LDF measurements of CBF in SAH patients monitored in the NICU. The concurrent capture of MRI and LDF data was achieved without compromising the data quality of either measurement, allowing for the simultaneous detection of both cerebral macro- and microcirculatory components. Both discrepancies and consistencies were found among the methods. This novel approach has the potential to provide comprehensive insights into global and local CBF, thereby increasing knowledge of the cerebral macro- and microvascular interplay in SAH patients.

Funding.

This work was supported by The Swedish Foundation for Strategic Research (RMX18-0056) and The Swedish Research Council (2020–03131).

Ethics approval.

This study was performed in line with the principles of the Declaration of Helsinki. The study was approved by the Ethics Review Board (EPN 2021–03527, 2021–08-02).

Consent to participate.

Informed consent was obtained from the participants’ next-of-kin since the participants were unconscious during the study.

## Funding

The clinical staff at the neurosurgery department (Linköping University Hospital) is gratefully acknowledged.

## CRediT authorship contribution statement

**Sofie Tapper:** Writing – review & editing, Writing – original draft, Visualization, Validation, Software, Methodology, Investigation, Formal analysis. **Stina Mauritzon:** Writing – review & editing, Writing – original draft, Visualization, Validation, Software, Methodology, Investigation, Formal analysis. **Marcelo P. Martins:** Writing – review & editing, Methodology, Investigation. **Fredrik Ginstman:** Writing – review & editing, Project administration, Methodology, Investigation. **Anders Tisell:** Writing – review & editing, Methodology, Investigation. **Peter Zsigmond:** Writing – review & editing, Supervision, Resources, Project administration, Methodology, Investigation, Funding acquisition. **Karin Wårdell:** Writing – review & editing, Supervision, Resources, Project administration, Methodology, Investigation, Funding acquisition, Data curation, Conceptualization.

## Data Availability

The data that has been used is confidential.
